# A Doctor Abroad

**Published:** 1873-10

**Authors:** 


HALL'S

### JOURNAL OF HEALTH.

VOL. XX.—OCTOBER, 1873.—NO. X.

### A DOCTOR ABROAD.

### A SUNDAY IN LONDON.

A BEAUTIFUL sunny morning in the later June. Most persons seem to be abed at eight o'clock; the streets are quiet and are almost deserted; here is a milkman, with a heavy can suspended by a contrivance from each shoulder, delivering each family its quota with an agreeable quietude compared with the rattling wheels over New York streets; but now and then the milk cart is also seen, most likely the Sabbath respecters requiring the footman's service. There are many rigid observers of the Sabbath day in London. A cabman was asked if he would call on Sunday for a drive to church; with deference he said, "I'm a Christian, sir, and do not drive on that day." Now and then a noisy newspaper vender was crying his wares, and an early cab with its solitary inmate, and a trunk on top, indicated the traveller. Newspaper offices, drug stores and provision shops were the only places open for business about Euston Square; a solitary woman was meekly drawing attention to the fact that she had watercresses for disposal at a cent a bunch.

At an early hour, by way of Blackfriar's Bridge, the cabman drew up before Spurgeon's "Tabernacle;" but learning that Mr. Summers would officiate in his stead, we passed a mile further on, and found welcome seats at "Surrey Chapel," where Rowland Hill had preached with faith, and power, and efficacy for near half a century, dying in his ninetieth year; his portrait was behind the pulpit, and at the right hand of it was the memorial stone of James Herman, who was the connecting link between the renowned dissenter of a past generation and the present famous incumbent, Newman Hall—who, like Henry Ward Beecher, needs neither prefix nor affix for designation, for there is but one of either on the globe to-day—nor in generations to come is it at all possible that their like will live, and work, and die in the Saviour's name, for His cause and kingdom.

The little quaint old brick building has been standing a hundred years, but the requirements of the times have caused plans to be laid to secure a new location near Westminster Bridge; this was foreseen eight years ago, when efforts began to be made to collect the funds needed for another site and a new building. These have been perseveringly continued until the authorities feel safe in encountering an expense of half a million of dollars in erecting a house which, in the language used to-day, "Shall be strong, continuous and useful," and, in another connection, should be "Beautiful, large and abiding." Three days ago the foundation stone was laid with songs of gladness, and tears of gratitude, and pæans of triumph, for the preparations have been made with hard work, in the face of envy and opposition. It would have been a gratification to many of the present, not to after generations, if the consecrated spot, with all its memorable associations, could have been held sacred to the same work for all time, but certain changes of population and business necessity made it expedient, in the estimation of far seeing Christian men, that the old ground should be abandoned. The Holy of Holies of ancient times had to change homes.

In entering the building, circular in form, with a lower floor, and a deep gallery all around, the mind is carried back to times far past—to ruder days, when necessities, not comforts, were the things to be considered.

THE PEWS

were hard boards, so narrow that if you did not sit up straight you would slide on to the floor; the aisles were barely wide enough for one person at a time, and just about as crooked as a monkey's big toe. Some pews had one, some two or more seats, thus adapting the capacity of the "sitting" to the varying length of the purses of the people. Some pews were straight, others were circular; then again, there were pews which were parallel to the pulpit in front and to the pulpit in the rear—others were diagonal, cata-cornered; then again, there were pews with a circular form and others with a circumbendibus which defied description. But this was not all. In the aisles there was a flap or a leaf board, attached to the aisle end of the pew with hinges, so that when the pews were full these could be raised, forming a seat. Thus the aisles were densely packed with attentive listeners to the great worker among the workers of London, with its three and three quarter millions of souls, reminding one of that indomitable worker in New York, the junior Tyng, who, like Newman Hall, not only has the will to work himself, but the admirable talent to make all who hear him both work and give, rendering the double service of the person as well as the purse—the former being more noble than the latter—for to sacrifice time and work for the Saviour's sake requires more of self-denial than the gift of a purse, a dollar, a guinea, or a ducat.

Americans may make a note of the pews here, and take advantage of the experience of a people growing up for a thousand years. Under the pew is a shelf, on which the hat may be placed, saving it from the dust of the floor. In the very handsome church of that good, and active, and efficient Presbyterian worker,

MORTON BROWN, of "Cheltenham," a far-famed watering place of England, there is a convenient slanting shelf in front of the sitter, with a rim on the edge, by which means a book may be laid open and secured from falling, without its being necessary to hold it all the time; thus the text can be kept always in sight. In some places there is another shelf, a few inches under the slanting one, upon which the hymn books may be laid. These are little things, but there is convenience in them. The selfish and unsocial system of having doors to pews is now going out of fashion; and, by general consent, the arrangement seems to be adopted that the pews are considered a right to the owner until the second hymn is sung, then, if not occupied, the sexton is at liberty to introduce "strangers." In Mr. Spurgeon's Tabernacle the aisles are broad enough to have a hinged seat on either side, with room for one person to pass when these are occupied, if the sitters face the minister; but if they face one another the aisle is completely blocked. To these seats strangers are directed, or they can go in and with perfect liberty take any one of them, thus preventing the tedious and uncomfortable standing at the door, and weary waiting for the sexton to show a seat, to be invited out by the tardy owner times not a few; and one may sit at his ease, enjoying his meditation, or looking around to see what is to be seen, the general understanding being that he is to feel perfectly at home until the sermon is about beginning, when he will have an opportunity of either remaining in his seat or of taking any vacant one to which the sexton may show him. This is perhaps the best plan, or very best arrangement which can be made in every house of worship where pews are rented or sold.

In many churches pews are of all sizes. In the Presbyterian Cathedral at Glasgow, and also where Norman McLeod officiated in his day, there are single pews, holding but one person—others two, many three, more over five or six. It is a great misfortune, in the United States, that in almost every church all pews are of the same size, compelling a family of two, when it cannot be really afforded, to rent a whole pew at a price no less than that which a *millionaire* pays; thus are many worthy Christian people excluded from the church of their choice, or they must be content to take cheap back seats, where, in their old age, with its impaired hearing, only a word now and then can be distinguished in those painful efforts at listening which takes away a great deal of the comfort of worship.

The sexton of the Glasgow Cathedral was understood to say that single sittings could be had there at a dollar a year, and he seemed to be greatly amazed that, in some of the New York Presbyterian churches, we paid for our pews fifty times that much for every sitting required for a family of "wife, children and friends."

CHURCH ON FIRE.

In the Marylebone Presbyterian Church of London, where Donald Fraser officiates, there are but two small windows in one end wall, some ten feet above the floor—all the others are in the roof; there is but one door of egress; the aisles are barely broad enough for two persons abreast; in case of fire, or any sudden alarm, the suffering and violent deaths which would be inevitable is horrible to think of. On leaving the church we said to a man who seemed to be one of the "pillars," "If this house should take fire you would all be roasted before your time." "Indeed!" was the laconic and impressive reply. We thought it not meet to prolong the conversation.

In some churches here, and also in America, there are hinged seats in part of the pews which face the pulpit. This ought to be the case in every meeting house in the world where the pews are rented. These should be considered free seats, where the old, and all others who hear imperfectly, and who are too poor to hire a seat, may come without let or hinderance; for such to be placed in seats farther from the pulpit, or in the gallery, is not less tantalizing to those who thirst for the word of life than to be famishing with thirst by the hour within sight and grasp of the bubbling spring, but with no power to quaff the delicious draught; and, inasmuch as the church has the poor and deaf always with them, such provision as suggested should be made in every house of worship, and not to do it is an inexcusable barbarism.

### "JEHU" LORDS.

Just as we were passing the field of Bannockburn, where Bruce defeated the English, and thus secured for himself the Scottish crown, a train of light colored cars whizzed along, known there as the train conveying Victoria the First; following thereafter there was another train for common mortals, a part of which was appropriated to the exclusive use of the son of the famed, and beautiful, and beneficent Dutchess of Sutherland, now dead—(this son thereby becomes the Duke of Sutherland)—who sat quietly reading a newspaper; a man of a common farmer look, tall, dressed in lightish woolen, looking for all the world like any other man, and seeming to be unconscious that there existed any thing on this planet than himself and his newspaper; they were all hastening to meet the Shah, who was to land on English soil the next morning, and to do this the Queen and the Duke had to travel all night. One wonders why they didn't leave earlier; but the Duke was leaving old Scotland, and the Queen her dearly beloved Balmoral, with the accent on the last syllable; thus both naturally put off the "bore," to the very last minute. When the Duke is in London the delight of his life and the very love of his heart is to "run to all the fires;" he takes as great pleasure in being present as any ten-year-old street urchin of New York; and day or night, if he is "in town," he is as certain to be at every occurrent fire as an eighteen year old belle is to be at a select party, where it is known that plenty of nice young men are to be present. To the end of not missing a fire the Duke's chamber is in telegraphic communication with every London district, and arrangements are made for giving him prompt intelligence of any noteworthy conflagration. This is one of the occupations of the grandees of this great realm of merry old England; but then there is horse racing, and fox hunting, and cricket, and base ball; but stage driving must not be left out. Some of the captains and "lords," and other young bloods of the kingdom, being demoralized and disgusted entirely with the impatient locomotive, which ruthlessly annihilated the old fashioned "coach-and-four" of a past generation, with all its picturesqueness and adventure, determined on "restoration;" and for the pure sentiment of the thing have organized a "line of stages." The programme runs thus: "The Richmond, Hampton Court and Tunbridge coach will run from the White Cellar, Piccadilly, London, at 11.30 A. M., returning at 6.45 P. M. Passengers and parcels carried at moderate rates, and punctually delivered." The drivers are titled men, all the way from captains in the army to lords, and perhaps higher still; of course, they only "take the reins" at the beginning of the journey of some twenty miles and back, and "hand them over" to a postilion at the end of the "drive." Although these things are of daily occurrence, there is always a crowd of gazers at the starting and all along the road. The street walkers and the country bumpkins stop and gaze with open mouths and wondering eyes, for there is a "lord" driving a stage. Store clerks, and butcher boys, and maidens all do not fail to run to the door, or hoist their chamber windows when the blast of the coach horn falls upon their ears from a distance. And it is rather a sight to see; for, in the first place, there is the "lord" and a "splendid team;" there, too, are all the passengers on the top of the vehicle—mostly "handsome young ladies," with their gay colored ribbons, and shawls, and streamers of every hue flying in the wind—and their faces are impressed with the consciousness of envied looks from multitudes of passers by, as much as to say, "I wish I had nothing to do but dress fine and ride on coach tops on sunny days, beside young lieutenants, captains and lords."

The "Jehu" always has a vacant seat beside him on the "off hand." This is "privileged," and each young lady hopes she will be the favored one, and be invited to that seat—especially American ladies, to whom it is "divine" to be at the side of a live young lord, and a handsome young fellow at that, and talk and chat for two whole hours. The most "taking" young damsel is sure to be selected, as the one who has the most mischief in her eye and is the most queenly in her bearing. And with what interest does she hang upon every word uttered, and how she follows the pointings of the finger, as he designates the various objects of interest along this classic ground, for there is some famous spot in almost every rod or two. Here Lord So-and-so lives; that is the residence of the Duke of R.; yonder the Duchess of T. holds her levees out of the Town; Louis Phillippe, the citizen king, passed his days of exile there, and died in that dumpy little brick building; there the Countess T. was born, destined in this year of the world, 1873, to be married to royal blood; there lived Alexander Pope, his garden stretching down to the very water's edge of the Thames; and on the opposite side of the road Horace Walpole, of famous memory, made his home. Two years ago the Duc D'Aumale lived inside that uncomely black brick enclosure—and yet within it is a most lovely home, with its flowers, and gardens, and orchards, and varied trees and evergreens; and last of all he comes to the very gate of Hampton Court, venerable for its age of some four hundred years, and memorable for the remarkable incidents which crowd around the place, from the inception of its plan, the laying of its foundation stone, its graceful uprising, its memorable change of ownership, and to its being the residence of a long line of royal personages, from Henry the Eighth, of famous memory, through the Jameses, and Charleses, and Williamses, and Georges within our own memories. But the glory has departed from Hampton Court; it belongs to the "Crown," but all the good Victoria gets of it personally is the annual product of that famous vine, planted over a hundred years ago, now a yard in circumference, with its twelve hundred and seventy bunches of grapes now pendant therefrom, dispensing for the Queen's table three thousand pounds a year, and a never failing crop, too.

In this famous "pile" are over sixteen hundred paintings of various kinds, many of them the portraits of the distinguished dead, and by artists whose names will never die. The far-famed Titian has given many originals. There remain the once splendid and memorable tapestries, now rich with the age of near four centuries—there as they were left hundreds of years ago—and the chambers, and beddings, and curtains, and canopies of kings and queens, who have long since mouldered into undistinguished dust; but there is not the scintillation of a remnant of the greatest man, taking him for all in all, who ever lived in the halls of Hampton Court,

OLIVER CROMWELL, who needs no title to tell in what order he was placed to designate his social or civil position, for he stood head and shoulders above all who had lived before him, and up to date, on English soil; he had no star, or "order," or ribbon; a plain countryman to look at, with his homely dress; no towering plume had he, nor gold or diamond fine; his work was that of the man, and that alone, and it was his genius that gave to England a glory which she never had before; and long will live, as among the greatest of the earth, the name of plain "Oliver," who times and oft rejected the offer of the name of "king."

To our eyes Hampton Court palace is one of the most beautiful places we ever beheld; its use now is a resort free for all who choose to go, and who conduct themselves with quiet and decorum; hatless and shoeless boys and girls were there, and many in garments all patched, and tattered, and torn, but clean. But another very commendable use is made of Hampton Court, with all its present beauties and its past grand memories; it is a "Home" for titled ladies, who were once wealthy and distinguished, but from whom fortune has departed, leaving them poor, indeed. Here they are provided for—the lady, the countess and the duchess—

some of them bordering on the hundred in age, owning some of the most famous names in history; all of them have some income of their own; none of them enough to secure a comfortable home, but enough to enable them to procure some little extra comforts. The tourist might well spend a week at Hampton Court.

